# From thought to action: The organization of spinal projecting neurons

**DOI:** 10.1016/j.celrep.2025.116153

**Published:** 2025-08-14

**Authors:** Carla C. Winter, Kuan Hong Wang, Zhigang He

**Affiliations:** 1 F.M. Kirby Neurobiology Center, Boston Children’s Hospital, Boston, MA 02115, USA; 2 Departments of Neurology and Ophthalmology, Harvard Medical School, Boston, MA 02115, USA; 3 Department of Neuroscience, University of Rochester Medical Center, Rochester, NY 14642, USA

## Abstract

Spinal projecting neurons (SPNs) are specialized neurons with cell bodies residing in the brain and axons extending into the spinal cord, providing a direct communication pathway that enables top-down control of nearly every bodily function. Disruptions to these pathways contribute to a wide range of neurological disorders, including developmental, degenerative, and traumatic pathologies. Advances in retrograde labeling, activity monitoring, and circuit manipulation have enabled increasingly precise and comprehensive characterizations of SPNs. Here, we provide a historical overview of brain-spinal cord connectivity research, followed by an in-depth synthesis of the current knowledge of SPN anatomical connections, molecular identities, and functional properties. We then propose a conceptual framework in which distinct SPN modules coordinately regulate motor, autonomic, and sensory processes to support bodily readiness and drive behavioral action. Beyond revealing the organizational logic of SPNs, these insights provide a foundation for designing therapies to restore brain-spinal cord communication following injury or disease.

## INTRODUCTION

We often take for granted that our thoughts seamlessly translate into actions. Yet, this remarkable ability relies on an intricate communication system between the brain and the body—a system that can be catastrophically disrupted by conditions like spinal cord injury (SCI), amyotrophic lateral sclerosis, or stroke, resulting in paralysis. Intriguingly, a similar paralysis-like state, known as motor atonia, occurs naturally during REM sleep, when the brain remains active while the body is rendered motionless, except for necessary respiration and eye movements. Whether pathological or physiological, paralysis involves more than just the loss of movement; it is also accompanied by altered sensory feedback and shifts in autonomic function. These striking parallels raise a fundamental question in neuroscience: how does the brain command the body?

**Figure 1.**
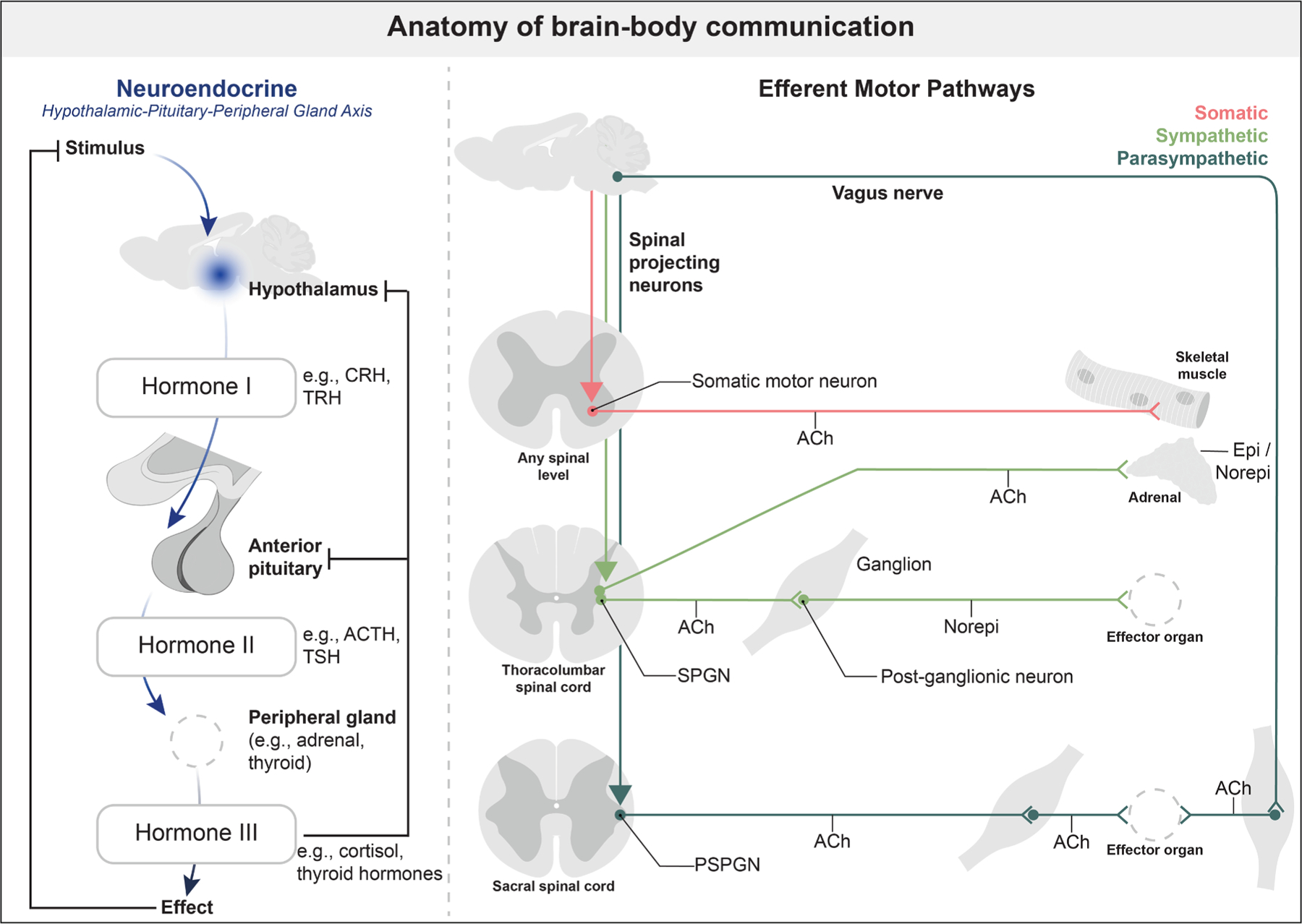
Anatomy of brain-body communication The brain commands the body via three primary routes. Left: the hypothalamic-pituitary-peripheral gland axis is a neuroendocrine system that allows the brain to directly influence, and be influenced by, peripheral targets. The hypothalamus, a part of the central nervous system, produces and releases hormones (e.g., CRH and TRH) into the pituitary portal circulation, which stimulate the release of hormones (e.g., ACTH and TSH) from the anterior pituitary gland. Anterior pituitary hormones are released into the systemic circulation and travel through the body’s circulation to impact distant organs (e.g., adrenal and thyroid). The hormones (e.g., cortisol and thyroid hormones) released by the peripheral glands can influence the hypothalamus and pituitary, creating a feedback loop that maintains appropriate hormone balances via bi-directional communication between the brain and the body. Arrows represent stimulation, and lines terminating in a vertical bar represent feedback inhibition. Right: the brain also directly influences bodily functions via the efferent somatic and autonomic motor pathways. The somatic motor pathway relays command signals from the brain to skeletal muscle. Here, spinal projecting neurons relay information from the brain, either via mono-synaptic or poly-synaptic connections, to somatic motor neurons throughout the spinal cord. The somatic motor neurons then relay cholinergic commands to peripheral skeletal muscle targets. The autonomic nervous system is broadly divided into two main pathways: the sympathetic and parasympathetic systems, which govern ‘‘fight or flight’’ or ‘‘rest and digest’’ bodily functions, respectively. The sympathetic nervous system is controlled by descending commands from spinal projecting neurons, which innervate SPGNs in the thoracolumbar spinal cord. These SPGNs send short cholinergic axons to synapse onto the sympathetic ganglia close to the central nervous system. The post-ganglionic neurons send long noradrenergic axons to peripheral targets. Some SPGNs directly innervate the adrenal medulla. The parasympathetic nervous system is controlled by descending signals that innervate PSPGNs in the brainstem or the sacral spinal cord. Both the brainstem and sacral PSPGNs send cholinergic axons to synapse with post-ganglionic neurons located in the ganglia close to their target organs, which in turn release ACh to modulate the function of the target organs. PSPGNs residing in the brainstem command peripheral targets via the motor component of the vagus nerve. CRH, corticotrophin releasing hormone; TRH, thyroid releasing hormone; ACTH adrenocorticotrophic hormone; TSH, thyroid-stimulating hormone; ACh, acetylcholine; Epi, epinephrine; Norepi, norepinephrine; SPGN, sympathetic preganglionic neuron; PSPGN, parasympathetic preganglionic neuron.

The answer to this question lies in three primary systems through which the brain commands the rest of the body: the endocrine system, the somatic nervous system, and the autonomic nervous system (Figure 1). The endocrine system operates through a cascade of chemical messengers released along the hypothalamic-pituitary-peripheral gland axis, which regulate a wide range of physiological processes independent of the spinal cord. In parallel, the somatic and autonomic nervous systems initiate actions primarily through descending brain projections that connect to peripheral targets via circuitry within the spinal cord. Somatic motor neurons located in the brainstem and spinal cord receive descending brain input and innervate hundreds of skeletal muscles, driving both voluntary and involuntary movements. The efferent arms of the autonomic nervous system, including both the parasympathetic and sympathetic branches, control countless smooth muscles, cardiac muscles, and glands, thereby orchestrating involuntary physiological processes such as heart rate, blood pressure, respiration, body temperature, and others. Both the sympathetic (i.e., ‘‘fight or flight’’) and parasympathetic (i.e., ‘‘rest and digest’’) nervous systems function via autonomic brain signals that stimulate preganglionic neurons located in the spinal cord. All sympathetic preganglionic neurons (SPGNs) are in the thoracic and upper lumbar spinal cord, while a subset of parasympathetic preganglionic neurons (PSPGNs) is situated in the sacral spinal cord. The remaining PSPGNs are in parasympathetic nuclei in the brainstem (e.g., dorsal vagal nucleus). As such, the spinal cord houses significant controllers of central nervous system outputs relevant to nearly all bodily actions. Notably, both somatic and autonomic motor outputs maintain a basal level of activity (or tone) that is dependent on the arousal state, and behavioral context further activates or inactivates these outputs.

It is also important to recognize that most behaviors are not solely driven by output signals. Instead, sensory information from the external environment and internal organs continuously modifies the activity of output neurons on a moment-to-moment basis. The dorsal horn of the spinal cord receives most afferent inputs from sensory neurons in the dorsal root ganglia (DRG). While such sensory information can directly regulate spinal output neurons, it can also be relayed to the brain through ascending pathways. Conversely, descending pathways can also directly modulate incoming sensory signals, for example, by decreasing incoming pain signals based on emotional- or survival-related needs. Together, incoming sensory information and outgoing brain commands from descending pathways form a closed-loop control system in regulating spinal cord function.

Taken together, the spinal cord serves as a key sensory processing center and houses both motor and autonomic output units, which grants it a degree of autonomy to execute basic physiological functions, particularly reflexive responses to peripheral stimuli. However, without commands from the brain, these functions often lack the contextual modulation necessary for generating purposeful behaviors. This is evident in the motor, sensory, and autonomic dysfunctions that arise following SCI and other conditions that disrupt brain-derived descending pathways. Indeed, patients with high cervical SCI often face survival challenges due to the extensive loss of integrated motor and autonomic control.^1,2^

Descending pathways are essential for transmitting high-level commands from the brain to the spinal cord, coordinating these ‘‘outflow’’ systems, and fine-tuning sensory processing during behaviors. A major component of these descending pathways is the spinal projecting neurons (SPNs)—a specialized population of neurons with cell bodies distributed across various brain regions and axons that project into the spinal cord. Due to the extraordinary length of their axons, SPNs are particularly susceptible to damage by body injury and brain trauma, such as in cases of SCI or stroke.^1,2^ In addition, the metabolic costs associated with building and maintaining such a long projection may put SPNs at risk in neurodevelopmental disorders such as hereditary spastic paraplegia^3^ and neurodegenerative diseases such as amyotrophic lateral sclerosis.^4,5^ On the other hand, the long distance covered by SPN axons also makes them potential therapeutic targets for a variety of neurostimulation techniques, including epidural spinal cord^6^ and deep brain^7^ stimulation.

Given their critical position in relaying brain-derived bodily commands and their unique features outlined above, decades of research on SPNs have investigated numerous intriguing questions in biology and medicine. How do these SPNs originate from different brain regions and project their axons all the way to the spinal cord? How do relatively small populations of SPNs exert such powerful command functions? How do they adapt to different behavioral repertoires? How do they respond to injury? Is it possible to regenerate their long projecting axons? In this review, we aim to synthesize both historical and contemporary research on SPNs using various technologies. We summarize different methods for classifying SPNs based on their anatomy (including brain origin, projection patterns, and spinal termination) and molecular features. Finally, we introduce an integrated framework to elucidate the functions of SPNs in behavioral contexts and shed light on the mechanisms by which thoughts are transformed into actions.

## HISTORICAL PERSPECTIVE

The interaction between the brain and body has long been a topic of interest in both philosophy and biology. The earliest known discussions about the brain’s role in controlling bodily functions date back to ancient Egypt, Mesopotamia, and Greece. The Edwin Smith Surgical Papyrus (circa 1600 BCE), an ancient Egyptian medical text, contains the first references to the brain, detailing head injuries and their effects on bodily functions, which suggests an early understanding of the brain’s involvement in movement and sensation.^8^ René Descartes (1596–1650) introduced Cartesian Dualism, proposing that the mind (a non-material entity) and the body (a material machine) interact primarily through the brain.^9^ He likened the body to a machine, with nerves acting as hollow tubes that carried ‘‘animal spirits’’ (fluid-like substances) from the brain to the muscles, enabling movement. This early concept foreshadowed later discoveries regarding neural pathways and reflexes.

During the early stages of modern neuroscience, advances in neuroanatomy and electrophysiological methods facilitated further functional analyses of brain-spinal cord connections. Charles Sherrington established that the motor cortex and brainstem could influence spinal cord function through pathways such as the corticospinal tract (CST), which carries voluntary motor commands, as well as the reticulospinal and vestibulospinal tracts in experimental animal models.^10^ In 1937, Penfield and Boldrey demonstrated that electrical stimulation of the primary motor cortex in human patients could elicit movement in specific body parts.^11^ Additionally, Claude Bernard conducted lesion and stimulation experiments on animals, showing that damage to specific brain regions, particularly the medulla oblongata, resulted in changes in blood pressure, indicating the brain’s control over autonomic functions.^12^ Together with observations from patients with SCIs and strokes, it became evident that different brain regions regulate various aspects of the spinal cord and bodily functions.

Despite these observations, anatomical evidence for SPNs was established much later, primarily due to their unique characteristics: sparse cell body distribution across diverse brain regions, long projecting axons extending to the spinal cord, and complex collateral branches targeting various subcortical structures. Early methods, such as the retrograde chromatolytic cell degeneration technique, provided some initial insights.^13^ In the 1970s, the introduction of neuronal tracers that could be transported through live axons in either anterograde or retrograde directions enabled detailed anatomical studies of long-projecting neurons like SPNs.^14,15^ These tracers allowed researchers to visualize individual axons along with their terminal arborizations and corresponding parent cell bodies. For instance, Hans Kuypers was the first to apply a retrograde tracer (horseradish peroxidase) to the spinal cord of cats, revealing a widespread distribution of SPNs across various brain regions.^16^ Various anterograde tracing methods facilitated examinations of the termination patterns of SPNs originating from individual brain regions within the spinal gray matter. Importantly, mapping the origins, projection trajectories, and spinal termination zones of SPNs has provided valuable insights into their potential functions. However, while these classical anatomical approaches offer valuable structural insights, they are inherently limited in both resolution and ability to assess functional properties.

## RECENT DEVELOPMENT OF VIRAL TOOLS

In recent decades, the advent of recombinant viral vectors offers unprecedented technologies to trace neural circuits, map synaptic connections, and manipulate gene expression within specific neuronal populations.^17,18^ These vectors differ in targeting directionality (anterograde or retrograde) and neuronal specificity (e.g., restricted to directly infected neurons or capable of crossing single or multiple synapses). Early studies demonstrated that rabies viral vectors could retrogradely label multiple brain regions when applied to peripheral organs, presumably via SPNs and their axons.^19,20^ However, because these vectors can traverse multiple synapses, the results do not definitively distinguish between direct and indirect connections between brain regions and peripheral organs. The development of recombinant rabies viral vectors overcame this limitation by deleting the gene encoding rabies envelope glycoprotein (G protein), which mediates transsynaptic viral transmission.^21–24^ By supplying the G protein in *trans* via a separate vector, this approach enables controlled viral propagation across a single synapse. Applying these vectors to various muscles not only directly labeled motor neurons in the spinal cord and brainstem but also revealed distinct premotor neurons in both the spinal cord and some brain regions, suggesting that certain SPNs directly innervate motor neurons.^25,26^ Similar efforts have also shown that several brain regions, particularly the rostroventral medulla (RVM), provide supraspinal presynaptic inputs to sensory afferents.^27^ Despite their utility in mapping monosynaptic connections, rabies-based vectors are limited by relatively low efficiency and neurotoxicity.

In contrast, the development of novel high-efficiency retrograde vectors has ushered in a new era in SPN research, enabling more precise and scalable mapping of brain-spinal cord connectivity. These vectors not only exhibit high efficiency but also have minimal toxicity and can deliver specific payloads to targeted populations of SPNs, facilitating cellular, molecular, and functional studies. The first of these was a pseudotyped lentiviral vector modified with the rabies virus glycoprotein, enabling retrograde targeting.^28,29^ When injected into the spinal cord, these vectors are taken up by axon terminals near the injection site. Pairing these vectors with an intersectional genetic approach, where a retrograde Cre recombinase is taken up by axons via injection into the spinal cord, and a Cre-dependent construct is delivered to the somata via injection in supraspinal regions, offers a targeted approach for functional manipulations. This approach has enabled the silencing of corticospinal neurons (CSNs) from different regions to assess their roles in fine motor control and sensory regulation through their descending spinal projections.^30,31^ A major limitation of this lentiviral vector is the difficulty in generating high-titer viral preparations.

More recently, several engineered adeno-associated viruses (AAVs) with robust retrograde transport capabilities, such as AAV2-retro, AAV9-retro, AAV2-MNM0048, and AAV11, have been developed through directed evolution and rational design.^17,32^ Depending on the specific region of the spinal cord into which these vectors are injected, they have been utilized to target termination-defined SPN populations for comprehensive anatomical, molecular, and functional studies. These studies have been conducted primarily in mice, but also in rats and non-human primates,^33^ and have already generated numerous new insights. The following sections will summarize the current understanding of SPN organization from anatomical, molecular, and functional perspectives.

## ANATOMICAL VIEW OF SPN ORGANIZATION

**Figure 2.**
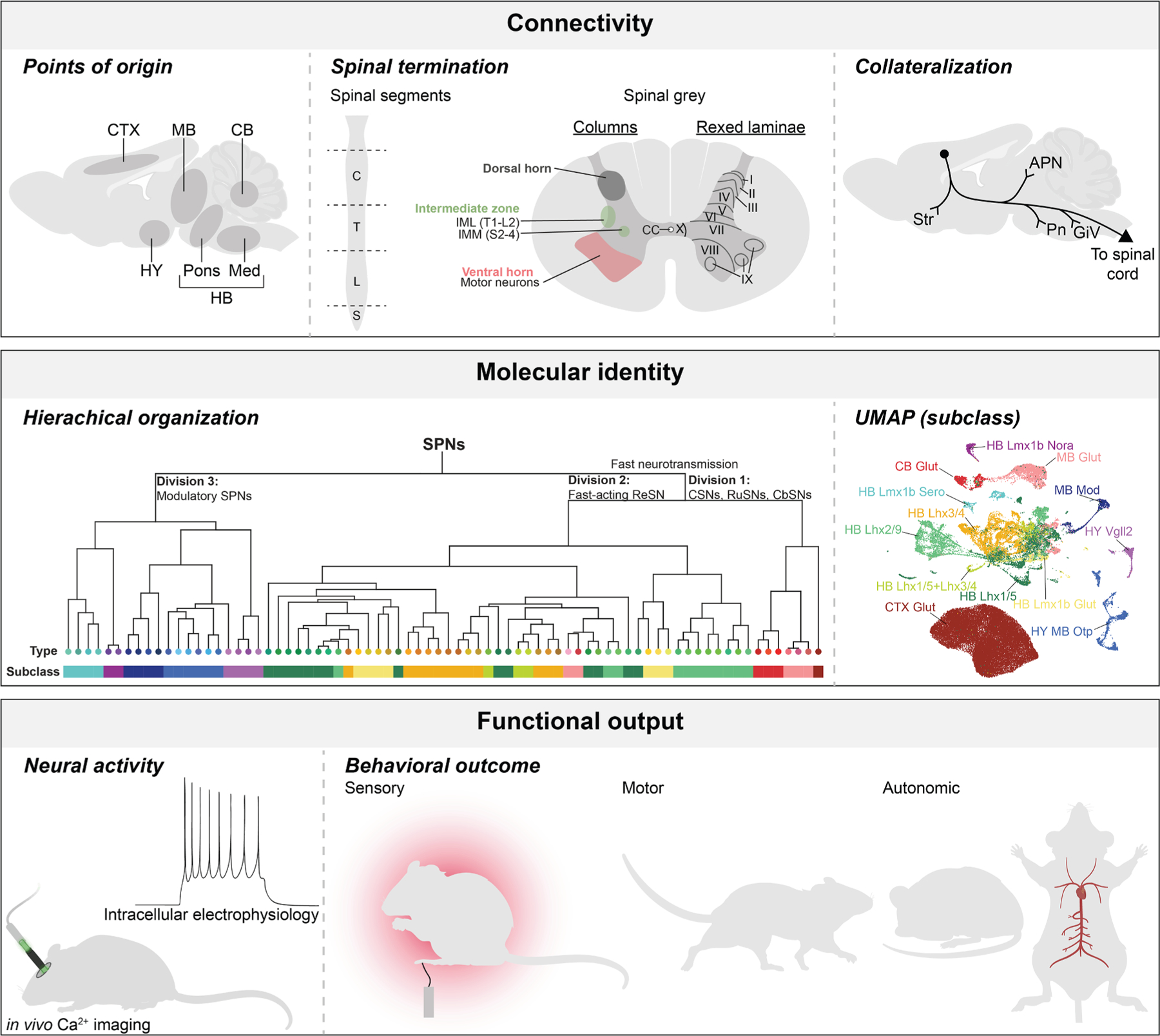
Approaches to defining spinal projecting neuron populations Historical and more modern approaches have enabled the characterization of SPNs across several domains. Top: first and foremost, SPNs can be defined by their connectome. Specifically, identifying their points of origin, spinal terminations, and axonal collateralization can offer a significant foundation for their functional roles. Their points of origin are throughout the brain, with most abundant populations in the CTX, HY, MB, CB, and HB. Their spinal terminations are defined by which rostral-caudal segment and gray matter zone their axons terminate in the spinal cord. Rostral-caudal segments are broadly divided into cervical, thoracic, lumbar, and sacral levels. Within each segment, the spinal cord gray matter is divided into three main gray zones (i.e., dorsal, intermediate, and ventral), which can be further subdivided into 10 Rexed laminae. SPNs may also be characterized by their axon collaterals, with many populations projecting to multiple target regions, enabling simultaneous control over multiple downstream circuits. Middle: SPNs may also be classified according to their molecular makeup. Single-nucleus transcriptomics has enabled high-throughput molecular characterization of brain-wide SPNs (detailed further in Figure 3). Images adapted from Winter et al., 2023. Bottom: finally, many efforts have sought to classify SPNs based on their functional outputs. Neural activity is primarily measured using *in vivo* Ca^2+^ imaging and intracellular electrophysiology. Behavioral analyses assess the contribution of SPNs to sensory (e.g., von Frey mechanical sensitivity test), motor, and autonomic systems. SPN, spinal projecting neuron; CTX, cortex; MB, midbrain; CB, cerebellum; HY, hypothalamus; Med, medulla; HB, hindbrain; C, cervical; T, thoracic; L, lumbar; S, sacral; IML, intermediolateral nucleus; IMM, intermediomedial nucleus; cc, central canal; Str, striatum; APN, anterior pretectal area; Pn, pontine nuclei; GiV, gigantocellular reticular nucleus ventral part; CSN, corticospinal neuron; RuSN, rubrospinal neuron; CbSN, cerebellospinal neuron; ReSN, reticulospinal neuron; Ca^2+^, calcium.

Since Kuypers’ seminal works, anatomical analysis has been considered as a key approach to understanding the function of SPNs.^34^ This understanding is based on the location of their cell bodies in the brain, as well as their projection trajectory and termination patterns in the spinal cord. As a subset of output neurons from individual brain regions, SPNs may convey region-dependent commands to the spinal cord (via their spinal terminations) and to other brain regions (via their collaterals). Thus, knowledge of the points of origin, projection trajectory, and spinal termination patterns of SPNs is highly informative for their functional outputs (Figure 2).

### Points of origin

It is reasonable to assume that the functions of SPNs correlate with the locations of their cell bodies. Recent retrograde tracing studies in mice and other species have revealed that over 30 distinct brain regions project to the spinal cord, albeit with varying cell numbers.^35–38^ Consistent with foundational studies,^39^ these modern dye- and viral-based tracing results identified numerous SPNs throughout the forebrain, midbrain, and hindbrain, with the largest population in the cerebral cortex (CSNs). Other prominent populations include those from the midbrain red nucleus (rubrospinal neurons [RuSNs]), deep cerebellar nuclei (cerebellospinal neurons [CbSNs]), and brainstem reticular formation (reticulospinal neurons [ReSNs]). Notably, large portions of the cerebral cortex (e.g., the temporal, occipital, and parts of the parietal and frontal cortices) and some regions with critical roles in motor output (e.g., the basal ganglia) do not project directly to the spinal cord. The logic underlying which brain regions contain SPNs and which do not remains unclear. Presumably, these regions lacking SPNs may relay information to other intermediary brain areas, which in turn transmit descending signals to the spinal cord via the brainstem.^40^

In general, reflexive and rhythmic movements are primarily managed by neurons in the spinal cord and brainstem.^41^ In contrast, goal-directed fine motor skills also depend on the critical functions of the cerebral cortex.^42^ Consistently, CSNs have been identified as a key substrate to transmit such cortical commands to the spinal cord. In most mammals, CSNs originate in a highly organized fashion from distinct areas: the primary and secondary motor cortex (M1 and M2 in rodents), the primary and secondary somatosensory cortex (S1 and S2 in rodents), and the cingulate cortex.^43,44^ These neurons are pyramidal cells mostly located in layer 5, characterized by tear-shaped somata and long apical dendrites extending toward the pial surface. In both M1 and S1 across species, CSNs controlling different body parts are arranged in a somatotopic manner. Neurons corresponding to the face, hand, arm, trunk, and leg are located progressively from lateral to medial along the motor cortex, and this spatial organization is preserved as the corticospinal fibers descend through the brainstem and spinal cord, effectively forming a ‘‘map’’ of the body within the CST, based on the body part each neuron controls.^39^ RuSNs in the red nucleus and CbSNs in the cerebellar deep nuclei share many functional and organizational features with CSNs, including their involvement in controlling limb movements and their somatotopic arrangement.^45,46^

ReSNs are another major population of SPNs that arise in the reticular formation of the midbrain and brainstem. Anatomically, the reticular formation refers to a diffuse, ‘‘net-like’’ structure comprising anatomical nuclei within the brainstem that are not well delineated by cytoarchitectural borders. Consistent with the wide range of functions attributed to the reticular formation, ReSNs have been implicated in diverse processes such as voluntary movement, involuntary postural and gait control, and autonomic regulation. Previous studies have shown that ReSNs are concentrated in specific regions such as the nucleus pontis oralis (PnO) and caudalis (PnC) in the ventral pons; the gigantocellular reticular nucleus (Gi), including its pars alpha (GiA) and ventralis (GiV), in the rostral medulla; and the ventral medullary reticular nucleus (MdV) in the caudal medulla.^35^ Recent studies using high-efficiency retrograde labeling have revealed that significant numbers of ReSNs are more widely distributed throughout the reticular formation than previously recognized.^36,37^ A spatially resolved transcriptomic atlas further suggests that these distributed populations may share common developmental origins, including the expression of LIM-family transcription factors.^38^

Other less abundant populations of SPNs originate from the forebrain paraventricular nucleus (PVN) and lateral hypothalamic area (LHA) of the hypothalamus; the superior colliculus and Edinger-Westphal nucleus of the midbrain; and the vestibular system, pontine micturition center (PMC, Barrington’s nucleus), and nucleus tractus solitarius (NTS) of the brainstem.^35–38^ Though points of origin yield some insights into SPN functional outputs, because most brain regions give rise to multiple projection pathways and support diverse functions, the anatomical origin of SPNs alone is insufficient to fully explain their functional roles.

### Projection trajectory and termination patterns

The spinal cord has a highly organized structure with rostral-caudally defined segments, dorsal-ventrally defined laminae, and medial-laterally defined areas. While all spinal segments host motor neurons in the ventral cord and receive sensory information at the dorsal horn, SPGNs and PSPGNs are located in the intermediate zone (IMZ) of the thoracolumbar and sacral spinal cord, respectively. Thus, the function of individual SPN populations can be partially predicted from their spinal projection level and termination patterns.

#### Spinal segments

As indicated above, an early indication of somatotopic cortical control of the motor function came from the pioneering work by Penfield and Boldrey, who showed that electrical stimulation of specific regions in M1 elicited movement in distinct body parts in humans.^11^ These results introduced the concept of a continuous ‘‘homunculus,’’ where body parts from head-to-toe map onto distinct regions of M1. Since then, numerous studies have shown that SPNs within M1 (i.e., CSNs) display somatotopic projections to specific spinal levels in both rodents^36–38,47–52^ and primates,^44,53,54^ suggesting their involvement in controlling movements of body parts corresponding to those spinal segments (e.g., projections to cervical, thoracic, and lumbar spinal cord command forelimb, trunk, and hindlimb movement, respectively). In addition, RuSNs and CbSNs also exhibit somatotopic organization, with neurons projecting selectively to forelimb or hindlimb regions of the spinal cord.^45,46^ In contrast, SPNs arising from many populations of ReSNs do not exhibit clear somatotopic segregation between cervical and lumbar projecting areas, though some regions, like the MdV, show a projection bias toward cervical segments.^36–38^

#### Medial-lateral projection

Intuitively, the SPNs projecting to the lateral or medial spinal cord may be more suitable for controlling asymmetric (limb) or symmetric (trunk) functions, respectively. Based on results from early studies, Kuypers and Holstege posited that descending motor pathways could be organized into three main groups: cortical, laterally projecting, and medially projecting pathways.^39^ In general, laterally projecting SPN populations are somatotopically organized and have few axon collaterals, allowing for precise, point-to-point control of specific spinal segments. Conversely, medially projecting pathways have extensive collateralization and little somatotopic organization, making them better suited for regulating posture, autonomic functions, and other broad spinal cord activities. In addition to this ‘‘voluntary somatic motor system’’ above, Kuypers and Holstege introduced the concept of ‘‘emotional motor pathways’’: descending pathways from supraspinal regions not evidently involved in motor control such as the hypothalamus, locus coeruleus, and caudal raphe.^55–57^

Although this framework remains generally valid, the specific SPN populations originally proposed are now known to be incomplete or imprecise, as newer anatomical results suggest that many ReSNs project to both the medial and lateral regions of the spinal cord. Moreover, the trajectories of some pathways vary across species. For example, although CSNs are functionally suited for segment-specific control, their projection trajectories differ across species: in rodents, CST axons travel through the dorsal column, whereas in primates (including humans), they descend via the dorsolateral spinal cord.

#### Termination patterns within the spinal cord gray matter

In each spinal segment, the output neurons and the key sensory processing centers are separately distributed, with the motor neurons in the ventral horn, SPGNs and/or PSPGNs in the IMZ, and sensory processing in the dorsal horn (Figure 2). In principle, SPNs can control downstream neurons in each of these distinct spinal gray zones through either direct (mono-synaptic) or indirect (di-synaptic or poly-synaptic) connections.

Although spinal motor neurons reside in the ventral horn, their premotor neurons are broadly distributed in the spinal cord, primarily in the ventral cord and IMZ.^58–60^ SPN populations influence motor output via synaptic connections to motor neurons and their premotor neurons. For CSNs, the degree of cortical control over spinal motor neurons appears to correlate with the evolutionary expansion of descending projections. This is reflected in the species-dependent termination patterns of CST axons: in relatively ‘‘primitive’’ species (e.g., opossums), CSNs terminate predominantly in the dorsal horn (associated with sensory modulation); in many other species (e.g., rodents and carnivores), they project to the IMZ, an integration center rich in premotor interneurons; and in primates, CSNs also reach the ventral horn, where they can form direct connections with motor neurons.^39^ Additionally, many ReSNs are likely mono-synaptically connected to spinal motor neurons, as shown by mono-synaptic rabies viral tracing, electrophysiological recordings, and histological analyses.^25^ Among these, inhibitory ReSNs in the medulla have been shown to directly innervate the somata of the motor neurons, potentially triggering hyperpolarization and contributing to muscle atonia during REM sleep.^61^

Several populations of SPNs are known to synapse onto the SPGNs and PSPGNs in the IMZ of the thoracolumbar and sacral spinal cord, respectively, to govern autonomic outflow. For SPGNs, neurons originating in the brainstem—for example, the excitatory and inhibitory rostral ventromedial medulla (rVMMe and rVMMi), PVN, and various modulatory nuclei such as C1 and A5—have been shown to innervate the IMZ directly and are likely to influence SPGN activity.^61–63^ These descending projections may terminate in different spinal segments and regulate subsets or the entirety of SPGNs via direct or indirect connections. While some of these pathways operate in the context of homeostatic regulation, such as the baroreflex, others are recruited under specific conditions, such as during a ‘‘fight-or-flight’’ response. On the other hand, SPNs in several brain regions, including those expressing corticotropin-releasing hormone (Crh) in the PMC,^64^ innervate the PSPGNs in the sacral spinal cord to regulate micturition and other physiological functions.

In parallel, various SPNs are known to make synapses onto neurons of the dorsal horn, which may either inhibit or facilitate the passage of incoming sensory information to the brain.^65–67^ Interestingly, these terminations exhibit certain laminae specificity. CSNs from the somatosensory cortex innervate the deep laminae of the dorsal horn, reflecting their role in modulating proprioceptive and tactile sensory processing.^31,68^ Notably, CSNs also terminate in the brainstem cuneate nucleus—a key relay for tactile and proprioceptive input—and modulate tactile feedback for the execution of dexterous movement.^69^ Other SPNs, including those from the anterior cingulate cortex, RVM, dorsal pons, and hypothalamus, preferentially innervate the superficial laminae of the dorsal horn, which receive and process nociceptive and mechanical sensory information.^61,70^ In addition to innervating the interneurons in the dorsal horn, these descending projections also target the pre-synaptic terminals of primary sensory afferents, forming ‘‘pre-synaptic inhibition.’’ Such top-down sensory modulation helps ensure the smooth progression of motor behaviors.^71–74^ Moreover, by selectively gating incoming sensory signals, descending SPN inputs may allow the spinal cord to contribute to attention, sensorimotor integration, and other cognitive functions.

#### Collaterals in the brain

Recent intersectional labeling techniques have enabled the visualization of SPN terminations along their entire descending pathways. Interestingly, some ReSNs also give rise to ascending collaterals, allowing them to influence supraspinal structures. For example, in addition to their spinal projections, SPNs in the rVMM send collaterals to innervate multiple nuclei in the medulla, pons, and midbrain.^61^ Within these projections, excitatory and inhibitory SPN populations share some targets but also exhibit distinct connectivity patterns. Both populations project to the laterodorsal tegmental nucleus (LDTg) and pedunculopontine nucleus (PPN), which contain cholinergic ascending neurons. However, excitatory SPNs mainly innervate motor-related cranial nuclei, including the facial nucleus, oculomotor nucleus, and trochlear nucleus, as well as PnO and MdV. In contrast, inhibitory SPN branches are more broadly distributed across brain regions involved in autonomic and sensory functions, including the NTS, locus coeruleus (LC), subcoeruleus nucleus ventral part, principal sensory trigeminal nucleus, A5 and A7 noradrenergic groups, periaqueductal gray (PAG), parabrachial nucleus, and spinal trigeminal nucleus. Notably, inhibitory SPNs avoid innervating the oculomotor and trochlear nuclei, which contain motor neurons controlling eye movements. These results suggest that these rVMM SPNs not only send commands to the spinal cord but also broadcast information to multiple supraspinal centers, enabling the coordination of motor, autonomic, and sensory functions across the central nervous system.

In addition, CSNs exhibit abundant collateral projections to a broad range of brain structures in rodents, including the striatum, anterior pretectal area, pontine nuclei, and GiV.^30,33,75–78^ Imaging studies of CSNs terminating in the striatum have shown that the activity of some of these neurons correlates with the onset and offset of forelimb movements, further supporting the broadcasting capacity of these long-projecting neurons.^78^ However, at least the collaterals of CSNs in the motor cortex appear more restricted in primates.^33^ Thus, despite different extents, the collaterals in the brain and the spinal cord are likely to be a general feature of SPNs, which might significantly contribute to their functional capacity.

### Synaptic inputs

Understanding the synaptic connectivity upstream of SPNs is critical to fully elucidating the circuitry required for brain-control of bodily functions. The use of modern viral-based transsynaptic labeling has enabled groups to investigate the whole-brain synaptic inputs that converge onto specific neuronal populations. Specifically, the aforementioned monosynaptic rabies virus system enables researchers to identify the synaptic input directly upstream of a neuronal population of interest. For example, rabies-based monosynaptic retrograde transsynaptic tracing was used to identify the inputs onto Crh^+^ SPNs arising in the PMC.^64^ They showed that these Crh^+^ PMC SPNs receive converging inputs from multiple brain regions, including three main classes: olfactory relay nuclei, cortical areas, and hypothalamic and brainstem nuclei. This complex set of projections to the Crh^+^ PMC suggests that it is an integrative center that uses olfactory and social information to regulate micturition. It should be noted, however, the percentage of all Crh^+^ PMC neurons projecting to the spinal cord is unknown. This study illustrates the utility of this type of brain-wide analysis of synaptic inputs to regions controlling the spinal cord. Extending this methodology into other spinal projecting populations will enable greater resolution of brain-wide efferent connections with descending pathways.

Together, differences in synaptic inputs, soma location, and axonal projection and termination patterns suggest that distinct SPN populations can relay specialized command signals to the spinal cord and other brain regions, thereby executing diverse control functions.

## SYNTHESIZING ANATOMICAL AND MOLECULAR FEATURES OF SPNs: A THREE-DIVISION FRAMEWORK

**Figure 3.**
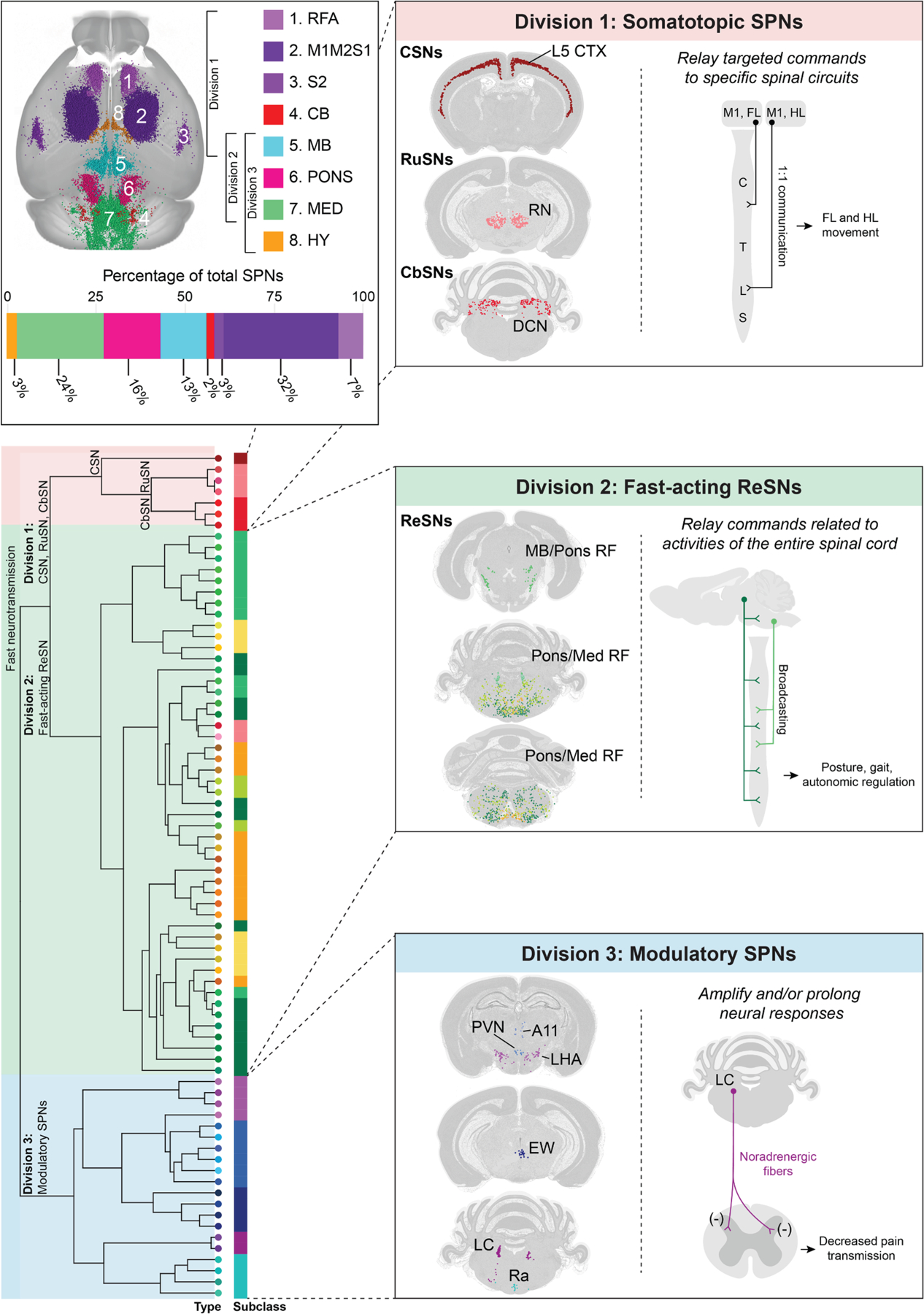
A three-division framework for spinal projecting neuron organization Synthesizing insights into the anatomical and transcriptomic identities of SPN populations yields a three-division framework for SPNs and informs how these neurons lead to diverse functional outputs. Left, top: adeno-associated virus-mediated retrograde labeling and whole-brain imaging of SPNs reveal that they arise throughout the brain, with most abundant concentrations in the CTX (M1M2S1, RFA, and S2), MED, PONS, MB, HY, and CB. The bar plot indicates the percentage contribution of each of the anatomically defined populations, rounded to the nearest digit. Left, bottom: single-nucleus transcriptomics of ∼65,000 retrogradely labeled SPNs across the whole brain revealed a hierarchical organization with 3 major divisions, 13 subclasses, and 76 cell types. The color blocks shading the taxonomy tree indicate division. The nodes at the end of the dendrogram indicate type, and the bar plots at far right indicate subclass. Right, top: division 1 of the SPN taxonomy corresponds to SPN types 1–7. Anatomically, MERFISH localizes these neurons to the L5 CTX, RN, and DCN (note: CSNs are in more restricted areas of L5 than indicated in the MERFISH results). These CSNs, RuSNs, and CbSNs each arise from discrete points of origin and have well-defined, somatotopically organized spinal projections. Molecularly, they are marked by relatively simple transcriptomes with discrete molecular markers and exclusively glutamatergic identity. These features suggest that they are suited for relaying commands to specific spinal circuits for ‘‘point-to-point’’ control of specific functions. Right, middle: division 2 corresponds to SPN types 8–56. MERFISH localizes these to sparse locations throughout the reticular formation. They comprise numerous transcriptionally defined cell types, have exceptionally complex transcriptomic expression patterns, and have glutamatergic, GABAergic, and glycinergic cell types. These anatomically and molecularly complex ReSNs with broad spinal termination patterns suggest that they are suited for relaying commands related to activities of the entire spinal cord, such as posture and autonomic functions. Right, bottom: division 3 corresponds to SPN types 57–76. MERFISH localizes these neurons to the hypothalamus (PVN and LHA), A11, EW, LC, and Ra. Molecularly, these neurons have unique neuro-modulatory properties, expressing slow-acting neurotransmitters (e.g., noradrenaline and serotonin) and/or neuropeptides. The release of these neuromodulators may allow these neurons to serve as a ‘‘gain setting’’ mechanism to amplify and/or prolong neural responses. For example, noradrenergic LC projections to the dorsal horn have been shown to decrease pain transmission. Images are adapted from Winter et al., 2023. RFA, rostral forelimb area; M1, primary motor cortex; M2; secondary motor cortex; S1, primary somatosensory cortex; S2, secondary somatosensory cortex; CB, cerebellum; MB, midbrain, MED, medulla; HY, hypothalamus; CSN, corticospinal neuron; RuSN, rubrospinal neuron; CbSN, cerebellospinal neuron; ReSN, reticulospinal neuron; L5, layer 5; CTX, cortex; RN, red nucleus; DCN, deep cerebellar nuclei; FL, forelimb; HL, hindlimb; C, cervical; T, thoracic; L, lumbar; S, sacral; RF, reticular formation; PVN, paraventricular nucleus of the hypothalamus; A11, cell group A11; LHA, lateral hypothalamic area; EW, Edinger-Westphal nucleus; LC, locus coeruleus; Ra, raphe nucleus; SPN, spinal projecting neuron.

In addition to anatomical features, the molecular properties of SPNs present a complementary perspective to their functional specification (Figure 2). Using retrograde tracers, previous work conducted bulk RNA sequencing to gain valuable insights into the molecular programs governing SPN development,^79–82^ axon targeting,^83–85^ and responses to injury.^86^ More recently, with the aid of highly efficient AAV vectors, single-cell resolution (i.e., single-cell and nucleus RNA sequencing) and spatial transcriptomic (i.e., MERFISH) approaches have enabled comprehensive brain-wide molecular profiling of SPNs, revealing the cell-type heterogeneity within spinal projecting pathways, even among neurons projecting within the same tract.^38,87,88^ Computational analyses of a single-cell resolution and spatial transcriptomic dataset of SPNs revealed a hierarchical organization with 3 major divisions, 13 classes, and 76 types.^38^ Interestingly, SPNs in these individual divisions are known to have unique anatomical properties. Unifying their anatomical and molecular features, a three-component organization of SPNs emerges, shedding light on how these characteristics may shape their unique functions (Figure 3).

### Division 1: Somatotopic SPNs

This division, identified through computational analysis, includes CSNs, RuSNs, and CbSNs.^38^ These neurons are exclusively excitatory and exhibit minimal to no expression of modulatory neuropeptides. Further, they are relatively transcriptionally homogeneous, with only a handful of molecularly defined cell types within each brain region and subtle molecular differences among these types. This feature is particularly striking for CSNs; although they are the most anatomically abundant population of SPNs and arise across distinct motor and sensory cortical regions (i.e., rostral forelimb area [RFA], M1, M2, S1, and S2), transcriptomic differences across these populations are subtle.

While these regions do not appear to contain multiple discrete CSN subtypes, differential gene expression analyses have identified subtle molecular distinctions between topographically defined CSNs. For example, genes such as *Epha4*, *Epha6*, *Epha7*, and *Efna5* are differentially expressed between cervical- and lumbar-projecting CSNs.^38^ These subtle molecular differences may reflect molecular cues required for establishing or maintaining soma topography and/or axonal targeting. Anatomically, CSNs, RuSNs, and CbSNs are characterized by somatotopically organized projections, originating from structured brain regions and terminating in spatially defined spinal segments. These anatomical and molecular features suggest that the functional specification of these SPNs may depend more on their synaptic connectivity, both in terms of origin and termination, than on molecular properties. For example, CSNs from M1 and S1 have similar transcriptional profiles but project to distinct spinal laminae, enabling them to govern separate functions in fine motor control and somatosensory processing, respectively.^31^ Together with their lack of high expression of modulatory neuropeptides, these neurons are particularly suited for relaying precisely targeted commands to specific spinal circuits for ‘‘point-to-point’’ control of specific functions.

### Division 2: Non-somatotopic, fast-acting ReSNs

This division corresponds to ReSNs expressing fast-acting neurotransmitters (i.e., glutamate, GABA, and/or glycine).^38^ These ReSNs comprise numerous transcriptionally defined cell types and have exceptionally complex transcriptomic expression patterns. Strikingly, unlike somatotopic SPNs in division 1, they are not easily definable by one or two marker genes; instead, they exhibit rich and complex transcriptional expression patterns with subtle distinctions among certain subtypes, reminiscent of the molecular complexity observed in non-SPN populations in the broader reticular formation.^89^ Further, these neurons are either excitatory or inhibitory, distinguishing them from the modulatory SPNs in division 3. Anatomically, many of these neurons project to multiple spinal segments, though not necessarily along the entire rostro-caudal extent of the spinal cord, which may underly their capacity in regulating spinal cord functions across different segments. For example, posture and gait control require the coordinated action of most spinal levels.^90^ Further, sympathetic regulation via these SPNs might engage most SPGNs across multiple spinal levels. Thus, unlike somatotopic division 1 neurons whose functions are tightly linked to spatial projection patterns, the molecular identities of division 2 neurons likely play a more prominent role in shaping their functional properties. Their combined molecular and anatomical heterogeneity likely underlies their exceptionally diverse roles in motor, sensory, and autonomic functions.

Of note, this division includes perhaps the most well-studied population of ReSNs: Chx10^+^ (Vsx2^+^) SPNs. Functional studies have heavily relied on Chx10 as a genetic marker of reticulospinal neurons,^91–94^ and strikingly, Chx10^+^ populations are involved in numerous motor activities, including locomotion,^95^ swimming,^96^ movement halting,^91,97^ and left-right turning.^93^ Their role in multiple distinct motor activities suggests that the Chx10^+^ class may comprise numerous subpopulations, underscoring a major limitation of using Chx10 as a single molecular handle for functional dissection. Further, unlike SPNs arising from well-defined anatomical nuclei, like the cortex and red nucleus, reticulospinal neurons arise across numerous nuclei largely without well-delineated anatomical borders.^35,98^ Given their overlapping spatial distributions, the use of a broad molecular marker like Chx10 is not sufficient to characterize different aspects of their functional repertoire and may yield confounding results. Indeed, single-cell resolution transcriptomic analysis have identified that Chx10+ neurons comprise at least 13 molecularly defined cell types.^38^

### Division 3: Modulatory SPNs

The last component of SPN organization includes populations throughout the hypothalamus, midbrain, and brainstem with unique neuro-modulatory properties, characterized by the expression of slow-acting neuromodulators like noradrenaline and serotonin or by a high abundance of neuropeptides. Early histochemical studies identified distinct neuropeptidergic and neuromodulatory populations in the hypothalamus, including neurons expressing arginine vasopressin in the PVN, α-melanocyte-stimulating hormone in the LHA and retrochiasmatic area, atrial natriuretic peptide in the perifornical hypothalamic area, and tyrosine hydroxylase (TH) in the dorsal hypothalamic area, among others.^99–101^ Modern transcriptomic studies have further expanded our understanding of these populations, identifying two main transcriptomic divisions—Otp^+^ and Vgll2^+^—corresponding to the PVN and LHA, respectively, each consisting of several molecularly distinct cell types.^38^ The midbrain contains two main anatomical regions with modulatory SPNs. The first is the A11 group of neurons that express TH, Dopa decarboxylase, and vesicular monoamine transporter 2. The second is the Edinger-Westphal nucleus, which contains both parasympathetic and non-cholinergic, centrally projecting neurons.^102^ Among these centrally projecting neurons are SPNs that express various neuropeptides, including cholecystokinin (Cck), cocaine- and amphetamine-regulated transcript (Cartpt), and urocortin (Ucn). Intriguingly, one Edinger-Westphal-derived SPN subtype appears to lack conventional markers of excitatory or inhibitory neurotransmission, instead expressing the aforementioned neuropeptides as its primary signaling molecules.^38,103,104^ The other large portion of these neurons is located in the hindbrain, with the most notable examples being the aforementioned noradrenergic and serotonergic neurons from the LC and raphe nuclei, respectively,^39^ each comprising several transcriptionally distinct cell types.^38,87^ Additional modulatory SPNs are distributed throughout the brain, including populations like glucagon (Gcg)-expressing SPNs in the NTS.

In theory, the co-release of slow neuromodulators and neuropeptides may serve as a ‘‘gain setting’’ mechanism to amplify and/or prolong neural responses, potentially enabling command signaling over extended time scales. This mode of signaling contrasts with that of division 1 neurons, which release exclusively fast-acting excitatory neurotransmitters, lack neuropeptides, and exhibit precisely targeted terminations, likely supporting spatially and temporally precise control. Moreover, the brain regions giving rise to divisions 1 and 3 are non-overlapping, reinforcing the notion that these divisions might represent distinct command signals derived from different brain regions for spinal control.

### Correspondence to previous works

This three-component organization of the spinal projecting system roughly aligns with classical theories of the motor system. The three divisions presented here parallel Kuypers’ and Holstege’s laterally projecting pathway controlling the independent movement of the extremities (division 1), medially projecting pathways controlling the axial and proximal body (division 2), and the emotional motor system involved in gain modulation (division 3).^57^ Although this classification is based on transcriptomic data from mouse SPNs, the divisions appear to correspond with their termination patterns, align with their general biological functions, and even help to overcome species-specific variations in projection patterns. For example, although CST axons primarily project in the dorsomedial spinal cord in rodents and the dorsolateral spinal cord in primates, their segment-specific termination patterns are conserved across species and support segment-dependent functions, such as digit movements and fine motor control. This classification further implies the existence of molecular codes that distinguish SPNs across divisions. Further studies should aim to identify the specific molecular programs that define each SPN division and determine whether SPNs in other species follow a similar or divergent organizational code.

### Why do ReSNs exhibit exceptional heterogeneity?

In principle, the molecular identities of SPNs may be shaped by both their developmental origins and context-dependent molecular adaptations aligned with their functional roles. Like the segmental organization of the spinal cord, the hindbrain develops from multiple segments called rhombomeres.^105,106^ However, unlike the relatively constrained migration seen in the spinal cord, progenitors in different rhombomeres undergo extensive migration within the compact hindbrain, resulting in individual hindbrain regions being composed of cells from multiple rhombomeres. This complex developmental mixing, followed by local adaptation to diverse regional cues and synaptic partners, likely contributes to the molecular diversity observed in ReSNs. Developmental transcription factors, particularly *Hox* and *LIM* genes, play critical roles in specifying the identity of each rhombomere. *Hox* genes show segment-restricted expression along the anteroposterior axis during development but have weaker or no expression during adulthood, while *LIM* genes also define specific anatomical regions during development but exhibit more persistent expression during adulthood, perhaps suggesting a role in maintaining neuronal identity beyond development. Supporting this, Cepeda-Nieto et al. identified three molecularly defined ReSN populations during development (Lhx1/5^+^, Lhx3/4^+^, and Lhx1/5^-^–Lhx3/4^-^) and showed that Chx10 (Vsx2) is expressed in a dynamic pattern within the Lhx3/4^+^ population.^107^ Remarkably, single-nucleus transcriptomic analyses of adult ReSNs also support a persistent *LIM* gene-based molecular code, which partitions pontomedullary ReSNs into five spatially segregated, LIM-defined populations^38^: Lhx3/4^+^, Lhx2/9^+^, Lmx1b^+^, Lhx1/5^+^, and Lhx3/4/1/5^+^. The Lhx3/4^+^ population shows near-complete overlap with the aforementioned Chx10^+^ ReSNs. Together, these findings suggest that transcriptomic profiles and developmental codes offer complementary and overlapping perspectives for defining the molecular identity and functional specification of ReSNs. The functional diversity and existence of multiple cell types within these broadly defined LIM groups emphasize the importance of considering the molecular diversity of ReSNs in functional studies.

Finally, it is interesting to consider that the molecular heterogeneity of ReSNs is a reflection not only of its developmental history but also of its evolutionary history. Compared with CSNs and RuSNs in division 1, ReSNs in divisions 2 and 3 are phylogenetically more ancient and exist across all vertebrates.^108^ As ReSNs evolved, diverse cell types may have emerged to adapt to environmental or physiological demands. Therefore, the molecular heterogeneity of ReSNs (and the reticular formation, more broadly^89^) may reflect a cellular ‘‘hard coding’’ of behavioral outputs, with their functional heterogeneity stemming directly from cellular diversity. This contrasts with the phylogenetically ‘‘newer’’ CSNs and RuSNs that have not had the same evolutionary time as ReSNs to develop additional cell types and are present in a more limited subset of vertebrates, with CSNs only present in mammals.^108^ Indeed, these neurons in division 1 comprise much fewer cell types with less molecular complexity compared with ReSNs.^38^ Therefore, the functional specialization of these cells seems to stem (or have evolved) from differences in connectivity rather than molecular makeup, which may arise through the combination of molecular cues and/or activity-dependent refinement during development. Interestingly, CSNs in division 1 exhibit an intriguing anatomical variation across species: direct corticomotoneuronal connections are only present in primates and are thought to be the substrates of enhanced manual dexterity.^2,109^ These direct corticomotoneuronal connections represent the purest form of ‘‘point-to-point’’ control and, intriguingly, are only present in the evolutionarily ‘‘newest’’ vertebrates.

## MODALITY-ORIENTED FUNCTIONAL INSIGHTS INTO SPN ORGANIZATION

With this foundational synthesis, this section will explore the role of specific spinal projecting populations in various bodily functions (Figure 2). As noted above, the spinal cord function has traditionally been divided into distinct modalities—motor, sensory, and autonomic—thus providing a convenient framework for exploring the functional outputs of SPNs. Early functional studies primarily relied on lesions or electrical stimulation of specific brain or spinal regions, with the resulting behavioral outcomes used to infer the function of the corresponding SPNs. More recently, advances in retrograde and intersectional labeling techniques have enabled targeted manipulation, including activation and inhibition, of defined SPN populations, as well as monitoring of their electrophysiological activity during behavior. These approaches are beginning to reveal new insights into SPN functions. In the following sections, we summarize selected examples from each of the three major modalities—motor, sensory, and autonomic—to illustrate how SPNs support behavior through dynamic and context-dependent control.

### Somatic motor outcomes

Somatic motor function refers to any bodily movement generated by skeletal muscles, ranging from complex forelimb movements like playing the piano or buttoning a shirt to rhythmic movements like walking and running.^110^ Each of these motor activities requires the coordinated activation of multiple muscles, spanning both gross motor skills (large-scale body movements) and fine motor skills (precise finger control). In addition to these voluntary components, motor behaviors are accompanied by involuntary adjustments that maintain posture, balance, and whole-body stability. In general, CSNs are thought to deliver goal-directed motor commands directly to the spinal cord. In parallel, subcortical SPNs—in particular, those arising from the brainstem—are primarily involved in reflexive and rhythmic motor behaviors such as locomotion^111^ and orofacial grooming.^112^ These subcortical SPNs may be also recruited to support and modulate goal-directed movements, providing an additional layer of motor control.^110,113^

Recent studies have begun to reveal how distinct SPN populations contribute to moment-to-moment control of movement. For example, using a battery of advanced circuit manipulation and monitoring approaches, Wang et al. proposed a model explaining the functional involvement of different subsets of CSNs in a skilled reaching and grasping task.^30^ First, *in vivo* imaging revealed that spatially segregated CSN populations are preferentially activated at different stages of task performance: gasping-related CSNs are enriched in the RFA in M2, while reach- and retrieval-related CSNs are mainly located in the caudal forelimb area (CFA) in M1 and S1. Consistently, ablating CSNs in the RFA impairs grasp-related movements, whereas CFA ablation disrupts reach-related aiming and advance movements. Both regions are required for wrist protonation, a transitional movement between reach and grasp. Upon optogenetic stimulation of CSNs, remarkable topographic joint activation patterns were observed: rostral-medial activation primarily elicits distal digit and wrist movements, while caudal-lateral activation mainly evokes proximal elbow and shoulder movements. The spinal termination patterns of these CSNs provide a plausible anatomical basis for this functional specificity. RFA CSNs primarily project to the intermediate laminae of the cervical spinal cord, where premotor neurons are located. On the other hand, CFA CSNs, particularly those from S1, preferentially innervate the deep dorsal laminae of the upper cervical cord, where tactile and proprioceptive afferents terminate. Thus, these findings suggest that CSNs may differentially regulate skilled motor behaviors by either controlling premotor circuits (RFA CSNs) or modulating sensory feedback and sensorimotor integration (CFA CSNs). In this way, distinct CSN populations form parallel and functionally specialized spinal projections, each contributing to specific phases of goal-directed motor skills. Importantly, CSNs do not act in isolation during this behavior. Many other subcortical SPNs, including RuSNs^114^ and ReSNs,^25,115^ have also been implicated in this skilled reaching task. These subcortical SPNs may receive input from CSN collaterals and/or from independent cortical pathways, forming a multi-layered control architecture that enables dynamic coordination of spinal cord activity during complex motor tasks.

### Sensory control

In addition to the descending input mediated by SPN axons, the other major source of input to the spinal cord is sensory information conveyed by DRG axons, which carry nociceptive, tactile, and proprioceptive signals from both peripheral targets and internal organs.^116,117^ These inputs lead to two major outcomes: they can either directly trigger intraspinal responses, forming the basis for classical sensorimotor reflexes, or be relayed to the brain via ascending pathways, informing the brain of the body’s peripheral state. Importantly, all stages of spinal sensory processing are subject to descending regulation by SPNs, potentially providing a neural basis for ‘‘mind over matter’’ control of the perception of pain. Some SPNs innervate the presynaptic terminals of sensory afferents through axon-axon interactions, modulating the volume of sensory input entering the spinal cord.^118^ Many others terminate on interneurons across different laminae, enabling filtering or tuning of sensory signals before they drive reflexes or ascend to the brain.^31,68,119^

Among these modulatory functions, descending control of nociception by brainstem-derived inputs, particularly from the RVM and PAG, has received considerable attention for its role in pain modulation and gating of nociceptive transmission in the dorsal horn.^120^ These areas contain neurons that are either activated or inhibited by noxious stimuli, referred to as Pain-ON and Pain-OFF cells, which are known to mediate stress-induced analgesia and produce analgesic effects upon direct electrical stimulation.^121,122^ Recent studies have identified specific SPN populations within the RVM, including rVMMe and rVMMi, that are involved in sensory, motor, and autonomic regulation.^61^ These neurons may overlap with the classically defined Pain-ON and Pain-OFF cells, participating in descending nociceptive control. Notably, a recent study uncovered an inhibitory descending pathway originating from the PMC, which exerts powerful control over nociceptive sensory input to the brain, further expanding the known circuits of descending pain regulation.^123^ Another well-known brainstem region directly controlling spinal pain pathways is the modulatory LC.^124,125^

In addition to brainstem-derived SPNs, CSNs in S1 have been shown to respond to tactile stimuli and regulate both normal touch sensitivity and neuropathic pain.^31^ Tactile signals from the periphery ascend through the dorsal column-medial lemniscus pathway to the ventral posterolateral (VPL) thalamus, which projects primarily to layer 4 of S1.^117^ Within S1, this input is relayed from layer 4 through layer 2/3 to reach layer 5 CSNs.^126^ Additionally, some VPL axons form direct synapses onto the dendrites of layer 5 pyramidal neurons, including CSNs.^127–129^ Through these pathways, CSNs in S1 integrate both ascending tactile signals and intracortical inputs, enabling them to participate in sensory-guided processing and exert descending control. Following peripheral nerve injury, mechanical allodynia (i.e., pain in response to light touch) can develop. Using retrograde labeling and *in vivo* imaging, tactile stimuli (e.g., brush or von Frey fiber) were found to activate a subset of S1 CSNs in intact mice, and this activity was significantly amplified after spared nerve injury (SNI).^31^ Chemogenetic silencing of S1 CSNs reduced both touch sensitivity in intact mice and tactile allodynia in SNI mice, demonstrating their role in both physiological and pathological sensory processing. Furthermore, optogenetic stimulation of S1 CSNs enhanced synaptic responses in CCK^+^ spinal interneurons, which relay light touch information from Aβ fibers. Co-stimulation of CSNs and sensory afferents led to super-additive synaptic responses and increased action potential output, indicating convergence and amplification of tactile signals. Together, these results reveal a spino-cortico-spinal sensitization loop, in which S1 CSNs directly modulate dorsal horn circuits to amplify tactile input, contributing to both normal sensation and mechanical allodynia. Thus, somatosensory CSNs may function as a top-down volume controller for touch and pain.

### Autonomic outflow

SPNs command both sympathetic and parasympathetic outflow of the autonomic nervous system. Similar to motor pathways, the effectors of autonomic outflow are located in the spinal cord (i.e., preganglionic neurons) but depend heavily on descending input for proper regulation. SPGNs regulate a wide range of physiological functions, including cardiovascular, respiratory, metabolic, and immunological activities, while sacral PSPGNs are primarily involved in bladder, sexual, and other autonomic functions related to the sacral spinal cord. Evidence for the critical role of supraspinal pathways in commanding and regulating autonomic outflow from these preganglionic neurons is seen in the occurrence of autonomic dysreflexia following SCI. While the crucial role of descending pathways in autonomic outflow is clear, the field is only just beginning to elucidate the specific contributions of distinct spinal projecting populations to autonomic functions.

#### Cardiovascular function

The sympathetic control of cardiovascular function is among the most extensively studied autonomic processes. Although classic brain lesioning studies implicated the medulla in maintaining basal blood pressure,^12^ the specific SPNs involved in top-down control remained unclear. Initial studies demonstrated a crucial role of the C1 adrenergic population in the lateral medulla in this process.^130^ However, further studies suggested that these neurons may be more important for responding to stressors such as hypoxia and ischemic insults.^131,132^ A more recent study suggested that SPNs in the rVMM directly innervate SPGNs and regulate blood pressure and heart rate in opposite directions, depending on their excitatory or inhibitory identity (rVMMe and rVMMi).^61^ Intriguingly, these SPNs can also coinnervate premotor neurons and even dorsal horn regions, suggesting a potential mechanism for the coordinated control of sensory, autonomic, and motor outputs during behavior. *In vivo* population recordings have shown that these SPNs are differentially engaged across arousal states. In the awake state, rVMMe SPNs are dominant. As a result, silencing these neurons leads to reduced sympathetic and motor tones, impairing high-speed locomotion. On the other hand, inhibitory rVMMi SPNs are the most active during REM sleep. Silencing these neurons disrupts sympathetic down-regulation and muscle atonia, suggesting a role in REM-associated motor suppression. Mechanistically, these rVMMi neurons have been shown to directly innervate spinal motor neurons,^61^ consistent with their ability to induce hyperpolarization and suppress motor output.^133–135^ Intriguingly, at least some of these rVMMi neurons also innervate the spinal dorsal horn, potentially overlapping with previously described pain-regulatory neurons.^120,122^

#### Other functions

Sympathetic activity regulates a wide range of physiological processes, including respiration, thermoregulation (through innervation of brown adipose tissue), immunity (through innervation of the thymus gland and spleen), metabolism (via projections to the gut and liver), and stress responses (through the adrenal gland), among others. SPNs may play critical roles in each of these functions via unique connections with SPGNs. For example, several studies have identified a descending thermos-regulatory pathway via projections from the rostral raphe pallidus to SPGNs, which controls non-shivering thermogenesis by brown adipose tissue.^136^ Additionally, it is plausible that SPNs are part of the efferent arm of the central inflammatory reflex^137^ via their connections to splanchnic SPGNs.^138–141^ Damage to SPNs may, therefore, provide a mechanistic explanation for central nervous system injury-induced immune deficiency syndrome^142^ seen in stroke, traumatic brain injury, and SCI. SPNs may also play a critical role in the brain-gut axis, for example, in mediating the effects of Gcg-like peptide (GLP)-1 on satiety and gut function. As mentioned previously, the medullary NTS contains a significant population of SPNs expressing Gcg, which produces preproglucagon that can be cleaved into GLP-1. These descending GLP-1 projections have been shown to preferentially innervate the SPGNs in the central autonomic area (i.e., Rexed lamina X) and the IMZ, which in turn project to the celiac, mesenteric, and pelvic ganglia, key regulators of gut function from the stomach to the rectum.^143^ Given this connectivity, the authors speculate that SPN-mediated GLP-1 input into SPGNs could modulate gut motility, secretion, absorption, and even blood flow, ultimately contributing to satiety and stress-induced hypophagia. This is a compelling example of how connectivity can predict function (i.e., SPNs to SPGNs to the GI tract) and how associated molecular information (i.e., GLP-1’s known role in satiety) further informs hypothesized functions.

#### Autonomic regulation

As many SPNs innervate SPGNs across multiple spinal levels, a key question is how specific patterns of sympathetic responses are achieved. Part of the answer may lie in the balanced interplay of regulatory mechanisms at multiple levels. First, in parallel to sympathetic pathways, the vagus nerve-associated parasympathetic system innervates many internal organs and co-regulates involuntary physiological processes, such as heart rate, blood pressure, and respiration.^144,145^ These parameters fluctuate around arousal state-dependent homeostatic set points, reflecting dynamic changes in basal sympathetic and parasympathetic tones.^146,147^ During heightened behavioral demands, such as physical exercise or the fight-or-flight response, sympathetic activity increases through allostatic regulation,^147,148^ supporting elevated skeletal motor output.^149,150^ Parasympathetic activity plays a critical role in restoring these set points during rest and recovery, maintaining cardiovascular stability and metabolic balance. By counterbalancing sympathetic drive, the parasympathetic system promotes physiological resilience through its support of rest, digestion, and energy conservation. Second, within the spinal cord, SPGN activity is regulated not only by descending supraspinal input but also by local spinal circuits, which are strongly influenced by sensory feedback. Third, beyond autonomic neural control, the endocrine system, particularly the hypothalamic-pituitary-peripheral gland axis, provides a complementary layer of longer-term physiological modulation, contributing to the regulation of stress, metabolism, and immune function.

## HOW CAN A SMALL SPN POPULATION COMMAND THE SPINAL CORD?

The results described above begin to shed light on key questions regarding the functional organization of SPNs. Notably, however, only a small fraction of brain neurons are SPNs, and SPNs constitute a similarly small proportion of presynaptic inputs to individual spinal neurons. This raises a compelling question: how can such a limited neuronal population exert such powerful and wide-ranging control over spinal function? Current evidence suggests that at least two key features of SPNs may underlie their command capabilities.

First, many SPNs appear to innervate multiple functionally distinct targets across both the brain and spinal cord. In principle, such broad projection patterns allow individual SPNs to recruit diverse end-effector organs, enabling coordinated activation of neural and physiological systems. As described above, CSNs in all mammals exhibit extensive collaterals in various subcortical structures and spinal areas. While CSNs in the motor cortex of primates reportedly have fewer collaterals than those of rodents,^33^ they still project to other brain regions such as the pons and interneurons in the IMZ of the spinal cord. Through these polysynaptic pathways, CSNs can influence not only motor output but also non-motor physiological outcomes. For instance, Peter Strick and colleagues demonstrated a descending pathway from M1/S1 and M2 cortical regions to internal organs (e.g., adrenal gland and stomach) through the sympathetic nervous system.^151,152^ Since many CSNs terminate in the IMZ, where SPGNs are located, this may represent a substrate for this cortex-to-internal organ connection. As mentioned above, in addition to M1 CSNs, CST axons from S1 terminate in the deep laminae of the spinal dorsal horn, likely modulating tactile and proprioceptive feedback,^30,31^ especially relevant for controlling proximal and axial musculature. These diverse projection patterns allow CSNs to participate in a broad range of motor functions, including action selection, movement initiation, and parameter tuning.^78,110,153^ Similarly, the intraspinal collaterals of ReSNs have long been recognized. As described above, SPNs from rVMMe and rVMMi innervate nearly all spinal levels, with shared terminations in the ventral horn, IMZ, and, specifically for inhibitory populations, the dorsal horn.^61^ This arrangement supports the integrated regulation of motor, sensory, and sympathetic tones. Additionally, these SPNs also innervate propriospinal circuits, thereby amplifying and/or multiplexing the descending command signals. Beyond the spinal cord, rVMM SPNs send ascending collaterals to innervate brainstem nuclei such as LDTg and PPN, where they may influence cholinergic ascending neurons and modulate cortical arousal states. These anatomical patterns suggest that SPNs are well positioned to mobilize multiple physiological systems in a coordinated fashion. However, it remains unknown whether all post-synaptic targets of SPNs are equally recruited during behavior or whether multi-target innervation is achieved by individual neurons or by functionally distinct subpopulations. Addressing these questions will require comprehensive mapping of both the inputs and outputs of individual SPNs, which may offer further insights into the regulatory logic underlying SPN-mediated control.

Second, the command capacity of SPNs may also be reflected in their complex molecular repertoire. It is noticeable that many SPNs co-express slow-acting neuromodulators, such as serotonin and TH-derived catecholamines, along with a wide array of neuropeptides. These neuromodulators and neuropeptides act primarily through G protein-coupled receptors, allowing them to amplify and prolong postsynaptic responses and to support signaling over extended time scales. Interestingly, these modulatory SPNs are mainly located in the hypothalamus, midbrain, and brainstem but are largely absent from the cortex, red nucleus, and cerebellum, where somatotopically organized, excitatory SPNs originate.^38^ This anatomical distinction suggests that command signals may vary in their spatial and temporal dynamics, ranging from precisely targeted to diffusely propagated modes of transmission. However, the extent to which these co-expressed, slow-acting neuromodulators and neuropeptides contribute to SPN-mediated command functions remains to be determined.

## A BEHAVIOR-ORIENTED VIEW OF SPN ORGANIZATION

**Figure 4.**
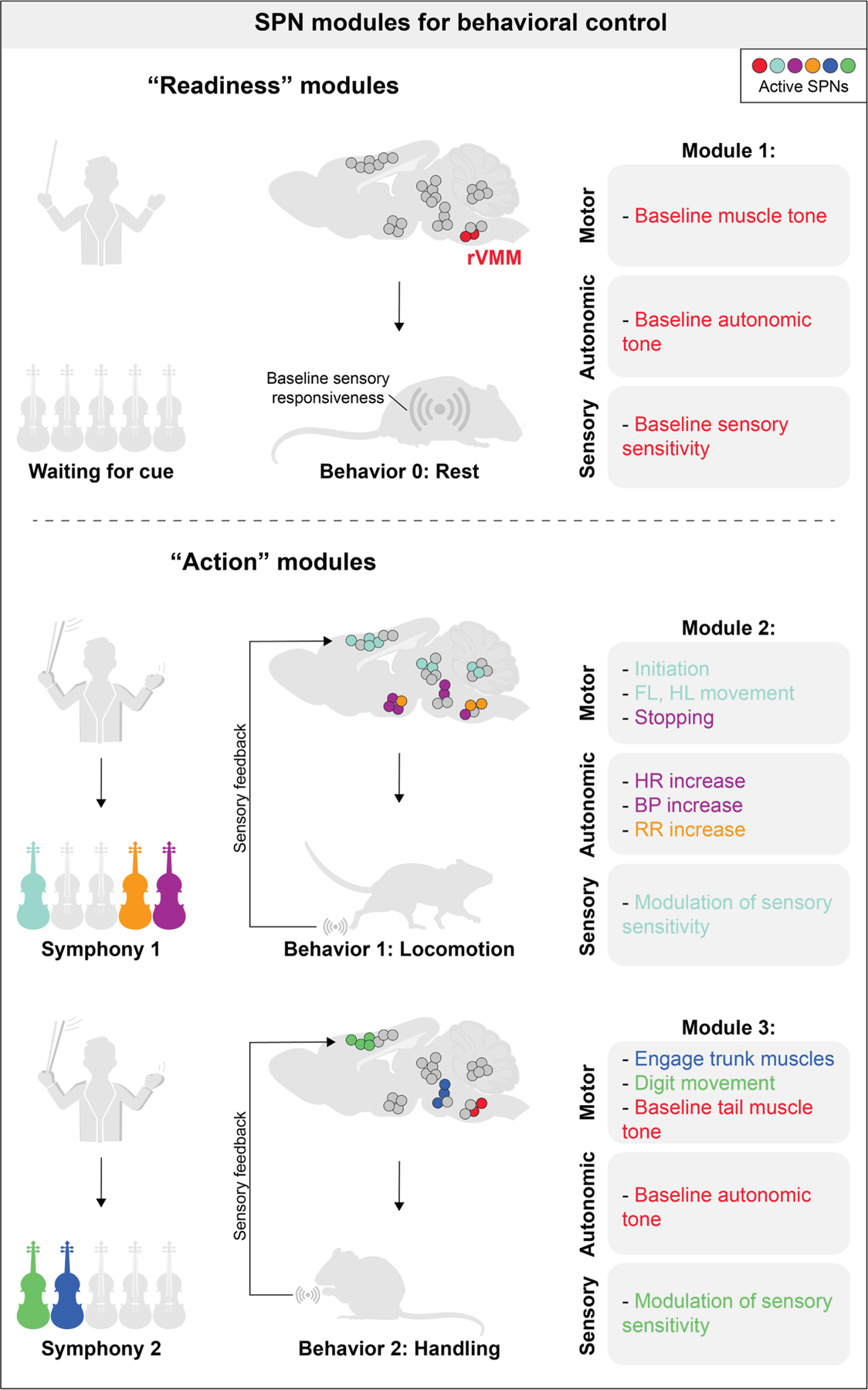
SPN modules control complex behaviors Spinal projecting neurons command complex bodily functions. Conceptually, these complex behaviors are the result of ‘‘modules’’ of motor, autonomic, and sensory functions, which, when executed together, generate unique behavioral outputs. These modules can be categorized into those supporting ‘‘readiness’’ and ‘‘action.’’ Top: ‘‘readiness’’ modules refer to a combination of motor, autonomic, and sensory states that prepares an organism to be able to perform a subsequent action. For example, an organism at rest has baseline muscle tone, sympathetic tone, and sensory sensitivity that prepares it to be able to perform a subsequent action. SPN populations (such as the rVMM, Zhang et al., Cell 2024)^61^ may control all three components of this readiness module. This readiness module is similar to an orchestra, with the conductor commanding the instrumentalists to be alert for the conductor’s subsequent cues to produce music. Bottom: ‘‘action’’ modules comprise a more complex set of motor, autonomic, and sensory functions that together generate complex behaviors like locomotion or handling. Action modules are likely the result of combinatorial recruitment of many different SPN populations throughout the brain. This is like a conductor who cues unique combinations of instrumentalists to produce distinct symphonies. SPN, spinal projecting neuron; rVMM, rostral ventro-medial medulla; FL, forelimb; HL, hindlimb; HR, heart rate; BP, blood pressure; RR, respiratory rate.

As described above, most studies so far have taken a bottom-up approach, inferring the behavioral involvement of individual SPN pathways based on their anatomical, molecular, and functional properties. This review now provides ample evidence to consider SPN organization from a top-down perspective—starting from behavioral demands to ask how different combinations of SPNs might dynamically coordinate to reconstitute moment-to-moment performance (Figure 4). From this vantage point, it becomes evident that nearly all behaviors require the real-time integration of motor, autonomic, and sensory modulation. For example, actions like walking or running demand not only the activation of patterned skeletal muscle activity but also autonomic preparation and regulation, including adjustments in respiration, heart rate, body temperature, and blood pressure. These physiological adjustments must be precisely tuned both before and during movement. Moreover, sensory feedback—including cutaneous, tactile, proprioceptive, visual, vestibular, and interoceptive signals—is critical for shaping and refining performance in real time. Despite appearing routine, even such seemingly simple behaviors require the coordinated contraction of large numbers of skeletal muscles (somatomotor), smooth and cardiac muscles (autonomic), and the processing of multiple sensory modalities.

In principle, individual behaviors involve the activation of many groups of SPNs in a coordinated manner. But how do these SPNs control and coordinate such diverse functions? There are two extreme possibilities: one possibility is that each SPN population innervates a specific target and executes a dedicated function. Somatotopically organized SPNs—such as CSNs, RuSNs, and CbSNs from division 1—may belong to this category (Figure 3). In addition to the somatotopic organization of their cell bodies in functionally distinct brain regions, their axons terminate in specific spinal segments and laminae, enabling precise motor or sensory modulation. An extreme case are the Betz cells in non-human primates and humans, a subset of CSNs that directly innervate specific sets of spinal motor neurons, allowing fine control of voluntary movement. The other possibility is that some SPNs broadcast signals to multiple targets in both the spinal cord and brain, recruiting behaviorally relevant neuronal populations that span multiple functional modalities (e.g., SPNs from divisions 2 and 3; Figure 3). As described above, this could be mediated by axonal collaterals and/or co-release of fast-acting neurotransmitters with slow-acting neuromodulators or neuropeptides. In this framework, such SPNs may serve as elemental control units or ‘‘syllables’’ of brain-to-spinal communication. Complex, sequential behaviors would then be generated by the combinatorial recruitment of distinct SPN populations, each contributing a discrete ‘‘melody,’’ much like an orchestra composed of multiple instrumental sections playing together to create unique symphonies. To define these control units, it is essential to understand the timing and pattern of SPN activity, as well as their conduction properties.

Conceptually, these behavioral control units might be categorized into those supporting readiness and those driving action. Readiness refers to the preparatory state of the organism, encompassing muscle tone for posture and gait control; sympathetic tone for cardiovascular, respiratory, and metabolic support; and sensory tuning for appropriate responsiveness to internal and external cues. Readiness is tightly linked to arousal state, emotional context, and motivational drives. In this context, it has been proposed that wakefulness is maintained by distinct arousal and action neurons, while sleep-related neurons are part of the central somatic and autonomic motor circuits.^154^ As described above, SPNs from the rVMM may be strong candidates for such readiness-related units.^61^ Some modulatory SPN populations that are relevant to arousal control might act similarly. Notably, readiness is not only essential for behavioral initiation but also continuously modulated throughout behavioral performance. Action-related units, in contrast, are likely to be more diverse, with contributions from many different SPN populations. Because multiple action-related units may serve overlapping or redundant functions, different combinations of SPNs can yield similar behavioral outputs. This flexibility and redundancy could underlie the robustness of motor and autonomic control and may prove critical in neural repair and recovery following SCI, stroke, or other pathologies.

Moreover, the composition and weighting of these units may vary across species and individuals, depending on their behavioral repertoires. For instance, bipedal vs. quadrupedal locomotion imposes distinct demands on postural and trunk stability, likely shaping the organization of SPN modules. Likewise, in primates, increased reliance on forelimb dexterity has driven the emergence of new SPN types, including those forming monosynaptic connections with spinal motor neurons to enable precise voluntary control.^2,109^ Together, these insights suggest that SPNs function as modular and adaptable control units, whose evolutionary and functional diversification underlies the brain’s ability to flexibly govern complex and context-dependent bodily actions.

## CONCLUDING REMARKS

How the brain controls the body remains one of the most fundamental and far-reaching questions in neuroscience. As explored in this review, SPNs are key pathways enabling the brain control of nearly every organ, tissue, and physiological process in the body. While both seminal work and more modern anatomical, molecular, and functional studies have significantly increased our understanding of these specialized neurons, our understanding of the spinal projecting system is far from complete.

A major remaining gap is understanding how distinct SPN populations are dynamically recruited during diverse behaviors (Figure 4). Future work should aim to clarify which populations are active during specific behaviors and how their activity patterns change across different tasks, internal states, or environmental contexts.

Additionally, although it is well known that damage to these pathways results in devastating dysfunction across motor, autonomic, and sensory systems, much more work is needed to understand their capacities for reorganization or repair after injury^155–158^ and how to harness this information to develop effective therapeutics spanning pro-regenerative strategies to brain-machine interfaces.^159^ One avenue to developing more effective therapeutics may be to better understand the natural phenomenon of heterogeneous injury response. Within an organism (e.g., mouse), different SPN populations appear to exhibit varying levels of plasticity and regenerative ability after injury. For instance, unlike the classically ‘‘regrowth-refractory’’ CSNs, subsets of ReSNs demonstrate appreciable regrowth and plasticity, which are thought to contribute to spontaneous locomotor recovery following SCI and stroke.^155,160–168^ Interestingly, the plasticity of different SPN populations after injury appears to parallel their evolutionary history, with ReSNs being the most primitive pathways and most appreciated for their capacity for plasticity following injury.^169^ Across species, there are also important differences in capacity for regeneration and functional recovery after injury.^170^ Understanding the cellular and molecular mechanisms underlying heterogeneous injury response may provide critical insights and lead to more effective treatments for regeneration and functional recovery.

Taken together, understanding these specialized neurons as a unified system coordinating movement, autonomic outflow, and sensory input provides deeper insights into how the brain commands the body and lays the groundwork for developing strategies for restoring that command when it is lost.
